# Knowledge, attitudes, and seizure first aid for childhood epilepsy among the Madinah community, Saudi Arabia

**DOI:** 10.3389/fpubh.2026.1858918

**Published:** 2026-09-21

**Authors:** Jawad Khalid Alsaedi, Salman Althobaiti, Nawaf Mohammed Alhejaili, Anas Nasser Alahmadi, Muhannad Saeed Aljazaeri, Ahmed Abdulrahman Alqarafi, Osama Abdullah Almohammdi, Yousef Abdullah Ghibban, Abdulrahman Nuwayfi Aloufi, Abdullah Faisal Aljohani, Rayan Hani Alhojilee, Ahmad Assinnari, Abdullah Alsarrani

**Affiliations:** 1 College of Medicine, Taibah University, Medina, Saudi Arabia; 2 Family and Community Medicine and Medical Education Department, Taibah University, Madinah, Saudi Arabia; 3 Department of Basic Medical Science, College of Medicine, Taibah University, Madinah, Saudi Arabia; 4 Life and Health Research Centre, Taibah University, Madinah, Saudi Arabia

**Keywords:** attitudes, awareness, epilepsy, knowledge, Madinah city, seizure

## Abstract

**Introduction:**

Epilepsy is one of the most common neurological disorders and affects people of all ages. In Saudi Arabia, its prevalence is higher than global estimates, with higher rates reported among children. Despite its prevalence, misconceptions and stigma surrounding epilepsy persist and may contribute to social exclusion, discrimination, and adverse psychological consequences, with children facing increased social challenges. Research on public knowledge awareness and attitudes toward childhood epilepsy remains limited in Al Madinah Al Munawwara. This study therefore assessed community knowledge awareness and attitudes toward childhood epilepsy and examined their associations with sociodemographic characteristics.

**Methods:**

A cross-sectional study was conducted among 580 adult residents of Al Madinah Al Munawwara between April and July 2025. Participants completed an online questionnaire assessing knowledge, awareness, attitudes, and seizure first aid knowledge. Descriptive statistics Mann–Whitney U and Kruskal-Wallis tests, Spearman correlation, and multivariable binary logistic regression were used for analysis.

**Results:**

Most participants had previously heard or read about epilepsy (87.3%), and attitudes toward children with epilepsy were generally favorable. However, important knowledge gaps were identified. Nearly two-thirds did not know what to do during a seizure (64.7%) or how to provide first aid to a child experiencing one (65.2%). Some participants reported inappropriate first-aid actions, including placing an object in the child’s mouth and restraining the child. Higher educational attainment and previous exposure to epilepsy were independently associated with better knowledge. Male participants had lower odds of good knowledge and favorable attitudes than females, while previous exposure to epilepsy was associated with more favorable attitudes. Knowledge awareness and attitudes were positively but weakly correlated.

**Conclusion:**

This study demonstrates that the Madinah community has high general awareness and favorable attitudes toward childhood epilepsy. However, practical knowledge of seizure first aid remains insufficient. Strengthening community-based educational initiatives may improve understanding and promote appropriate responses to seizures.

## Introduction

Epilepsy is a neurological disorder characterized by a persistent predisposition to recurrent unprovoked seizures and may arise from structural, genetic, infectious, metabolic, immune, or unknown causes (1). It is one of the most common disorder and affects an estimated 50 million people worldwide, with active epilepsy occur in 4–10 per 1,000 population (2). In Saudi Arabia, its prevalence is estimated at 6–8 per 1,000 population, with higher rates reported among individuals under 18 years of age than in other age groups (3, 4). Despite its prevalence, misconceptions and stigma surrounding epilepsy persist and may contribute to discrimination, social exclusion, and negative psychological and social consequences for people with epilepsy (5, 6).

Studies from Saudi Arabia have identified important gaps in public knowledge and attitudes toward epilepsy. A study conducted among parents in the Al-Baha region found that over 90% had limited knowledge of the causes of epilepsy and held misconceptions about the condition (7). Another study of more than 7,000 participants from five regions of Saudi Arabia also reported gaps in knowledge, with misconceptions about the causes and course of epilepsy remained common alongside negative attitudes toward social interaction, marriage, and employment involving people with epilepsy (8). These findings highlight the social and psychological consequences that people with epilepsy may face due to such misconceptions and negative attitudes.

Focusing on childhood epilepsy is important because the condition can affect children’s development, education, psychological wellbeing, and social life (9). Caregivers and community members also play an important role in recognizing seizures, providing appropriate first aid, and supporting children with epilepsy at school and in social settings. Given the prevalence of childhood epilepsy and misconceptions about the condition in Saudi Arabia, community perceptions and attitudes toward children with epilepsy and the factors associated with them need to be better understood. Research in this area remains limited in Saudi Arabia. Al Madinah Al-Munawwara is known for its diverse population and receives visitors from different cultural backgrounds, which may contribute to variation in perceptions and attitudes toward epilepsy. Previous studies conducted in Al Madinah have focused on specific groups, such as school teachers (10) and university students (11), rather than the wider community. Therefore, this study aimed to assess community knowledge, awareness, and attitudes toward childhood epilepsy in Al Madinah Al-Munawwara, evaluate knowledge of appropriate seizure first aid, and examine their associations with sociodemographic characteristics.

### The objectives of our study

1. To assess the Madinah community’s knowledge, awareness, and attitudes toward childhood epilepsy.
2. To evaluate the Madinah community’s knowledge of appropriate seizure first aid.
3. To examine associations between sociodemographic characteristics (nationality, age, gender, marital status, educational level, and monthly income) and the community’s levels of knowledge, awareness, and attitudes toward childhood epilepsy.

## Methodology

### Study design

This descriptive cross-sectional study was conducted in Al Madinah Al Munawwara, the provincial capital of the Al-Madinah region, in western Saudi Arabia. Data was collected between April and July 2025. The study aimed to assess the knowledge, awareness, and attitudes toward childhood epilepsy among residents of Al-Madinah Al Munawwara.

### Study population

The target population included adult residents of Al Madinah Al Munawwara. Participants were eligible if they were aged 18 years or older and residing in Al Madinah Al Munawwara at the time of the study. Individuals younger than 18 years and those who did not reside in Al Madinah Al Munawwara were excluded.

### Sample size

The sample size was calculated using Raosoft online software, applying a standard sample size formula based on predefined statistical parameters. Considering a standard deviation of 1.96 for the 95% confidence interval and a maximum acceptable margin of error of 0.05, the minimum required sample size for this study was determined as follows:

$$n=\frac{{\left(1.96\right)}^{2}\times 0.50\times 0.50}{{\left(0.05\right)}^{2}}$$

Therefore, with an estimated population of 2,137,000 in Madinah, the calculated minimum sample size required for this study is 385 participants.

### Sampling and data collection

A convenience sampling technique was used to recruit participants from the community. Data was collected using an online questionnaire hosted on Google Forms and distributed through WhatsApp and other social media platforms. Google forms was set to accept only one response from each participant. Ten volunteers residing in Al Madinah Al Munawwara assisted with distribution by sharing the questionnaire with their contacts. Participants received information about the study objectives before completing the questionnaire, and electronic informed consent was obtained before participation. The questionnaire took approximately 5 to 10 min to complete. In line with the CHERRIES guidelines for reporting results of internet surveys (12), and all questionnaire items were mandatory, with responses submitted only after all required items had been completed. No financial incentives were offered for participation. Because the questionnaire was distributed through private messaging and social media, the number of individuals who received or viewed the invitation could not be determined. Therefore, view and participation rates could not be calculated.

### Questionnaire development

A structured questionnaire was developed based on a review of similar published studies assessing knowledge, awareness, and attitudes toward epilepsy (13–16). The questionnaire was translated into Arabic. The questionnaire consisted of 34 questions divided into four sections. The first section collected demographic information, including age, gender, marital status, education level, and monthly income. The remaining sections included 24 questions assessing awareness, knowledge, and attitudes toward childhood epilepsy.

### Pilot study and instrument assessment

Before data collection, the Arabic questionnaire was pilot tested among 10 participants to assess the clarity and comprehensibility of the questions and identify ambiguous items. Following discussion within the research team, a small review committee was formed to evaluate the pilot feedback. The committee comprised two independent researchers from the College of Medicine at Taibah University with background in pediatrics and neurology and one coauthor with background in community medicine. The committee reviewed the questionnaire and pilot feedback for relevance, clarity, and appropriateness, and changes were made where necessary. Internal consistency of the knowledge awareness and attitude domains was explored in the pilot sample using Cronbach’s alpha. Participants involved in the pilot study were excluded from the main study.

### Statistical analysis

All statistical analyses were conducted using IBM SPSS Statistics version 30. Descriptive statistics, including frequencies, percentages, means, and standard deviations, were calculated to characterize the sample and key study variables. All scores for knowledge, awareness, and attitude were standardized to a scale ranging from 1 to 100 to facilitate comparison across domains. The mean scores were 46 for knowledge, 49 for awareness, and 88 for attitude, and these values were used as cutoff points to dichotomize participants into good versus poor categories, with scores at or above the mean classified as good and those below the mean as poor. This mean-based dichotomization approach was adopted from similar studies classifying knowledge awareness and attitude scores (17–19). This approach was used to classify participants relative to the distribution of scores within the study sample.

For knowledge questions with multiple correct responses, answers were considered correct if the participant identified at least half of all correct options without selecting the “I don’t know” response. Prior to inferential testing, data were assessed for normality using the Shapiro–Wilk test and visual inspection of histograms and Q-Q plots. Because the data violated normality assumptions, non-parametric tests were employed for group comparisons.

Mann–Whitney U tests were used to compare two independent groups (nationality, gender, and exposure), while Kruskal-Wallis H tests were applied to comparisons involving three or more groups (age, marital status, education level, and monthly income). To identify independent predictors of knowledge, awareness, and attitudes while controlling for potential confounders, three separate binary logistic regression models were constructed with demographic and exposure variables entered as predictors. Model fit was evaluated using the Hosmer-Lemeshow goodness-of-fit test, and the variance explained was assessed using Cox and Snell R^2^ and Nagelkerke R^2^ statistics. Statistical significance was set at *p* < 0.05 for all analyses.

### Ethical considerations

The study objectives were explained to all participants, and informed consent to participate was obtained before answering the questionnaire by selecting the ‘YES’ checkbox. Alternatively, they could choose the ‘NO’ option if they decided not to participate. This research received approval from the Research Ethics Committee at Taibah University, Al-Madinah Al-Munawwara; the study ID was STU-25-018.

## Results

The study sample consisted of 580 participants, predominantly Saudi nationals representing nearly 90% of the sample. The age distribution showed that nearly half were young adults aged 18–30 years. Females constituted a slight majority at 56%, and just over half of the participants were married. Educational attainment was relatively high, with nearly half holding bachelor’s degrees and an additional 7% having master’s or higher qualifications. Income distribution varied, though more than a third of participants preferred not to disclose their income level (Table 1).

**Table 1:** Sociodemographic characteristics of participants (*N* = 580).

Category	n (%)
Nationality	
Saudi	518 (89.3%)
Non-Saudi	62 (10.7%)
Age group	
18–30 years	273 (47.1%)
31–45 years	132 (22.8%)
46–60 years	147 (25.3%)
>60 years	28 (4.8%)
Gender	
Female	324 (56.2%)
Male	253 (43.8%)
Marital status	
Single	251 (43.5%)
Married	312 (54.1%)
Divorced/Widowed	14 (2.4%)
Educational level	
Elementary or below	11 (1.9%)
High school	185 (31.9%)
Diploma	75 (12.9%)
Bachelor’s	267 (46.0%)
Master’s or above	42 (7.2%)
Monthly income (SAR)	
<3,000	134 (23.4%)
3,000–7,999	54 (9.4%)
8,000–14,999	89 (15.6%)
>15,000	90 (15.7%)
Prefer not to say	205 (35.8%)

The median knowledge score was 46.15 (IQR 30.77–61.54), while the median awareness score was 40.00 (IQR 40.00–60.00). The median attitude score was 100.00 (IQR 83.33–100.00) (Table 2). Internal consistency coefficients from the pilot assessment are presented in Supplementary Table S1.

**Table 2:** Distribution of knowledge, awareness, and attitudes scores.

Statistic	Mean (SD)	Median (Q1–Q3)
Knowledge	45.98 (18.40)	46.15 (30.77–61.54)
Awareness	49.31 (22.34)	40.00 (40.00–60.00)
Attitude	87.64 (16.70)	100.00 (83.33–100.00)

Most participants reported familiarity with epilepsy and recognized that it is not contagious. Gaps were observed in knowledge of the causes of epilepsy and seizure management. Around two-thirds reported that they did not know what to do during a seizure or how to provide first aid to a child experiencing a seizure. More than half had previously encountered someone with epilepsy, although attendance at epilepsy awareness campaigns was low. Most participants expressed interest in attending an awareness session (Table 3). Among the reported first aid responses seeking medical help and keeping the child safe from nearby objects were the most frequently selected actions. Some participants also selected inappropriate actions such as placing an object in the child’s mouth or restraining the child (Figure 1).

**Table 3:** Knowledge and awareness about epilepsy (*N* = 580).

Item and response	n (%)
Do you know what epilepsy is?	
No	104 (17.9%)
Yes	476 (82.1%)
Do you believe that epilepsy is a lifelong illness?	
No	147 (25.3%)
Yes	205 (35.3%)
Do not know	228 (39.3%)
Do you think epilepsy is a serious disease?	
No	134 (23.1%)
Yes	343 (59.1%)
Do not know	103 (17.8%)
Do you believe that epilepsy is a contagious disease?	
No	548 (94.5%)
Yes	4 (0.7%)
Do not know	28 (4.8%)
Do you believe that epilepsy is a hereditary disease?	
No	170 (29.3%)
Yes	250 (43.1%)
Do not know	160 (27.6%)
Do you believe that epilepsy is a mental illness?	
No	252 (43.4%)
Yes	186 (32.1%)
Do not know	142 (24.5%)
Do you believe that epilepsy is a curable disease?	
No	71 (12.2%)
Yes	382 (65.9%)
Do not know	127 (21.9%)
Causes known	
No	430 (74.1%)
Yes	150 (25.9%)
Triggers known	
No	291 (50.2%)
Yes	289 (49.8%)
Manifestations recognized	
No	201 (34.7%)
Yes	379 (65.3%)
Knowledge of what to do during seizure	
No	375 (64.7%)
Yes	205 (35.3%)
How effective is medical treatment for epilepsy?	
Ineffective	24 (4.1%)
Sometimes effective	259 (44.7%)
Do not know	200 (34.5%)
Always effective	97 (16.7%)
Do you know how to provide first aid to a child having an epileptic seizure?	
No	378 (65.2%)
Yes	202 (34.8%)
Have you ever heard or read about epilepsy?	
No	73 (12.7%)
Yes	504 (87.3%)
Do you have a family member or close friend who suffers from epilepsy?	
No	430 (74.1%)
Yes	150 (25.9%)
Have you ever encountered someone with epilepsy?	
No	248 (42.8%)
Yes	331 (57.2%)
Have you attended any awareness campaigns or events about epilepsy?	
No	512 (88.3%)
Yes	68 (11.7%)
Would you like to attend an awareness session about epilepsy?	
No	203 (35.0%)
Yes	377 (65.0%)

**Figure 1:**
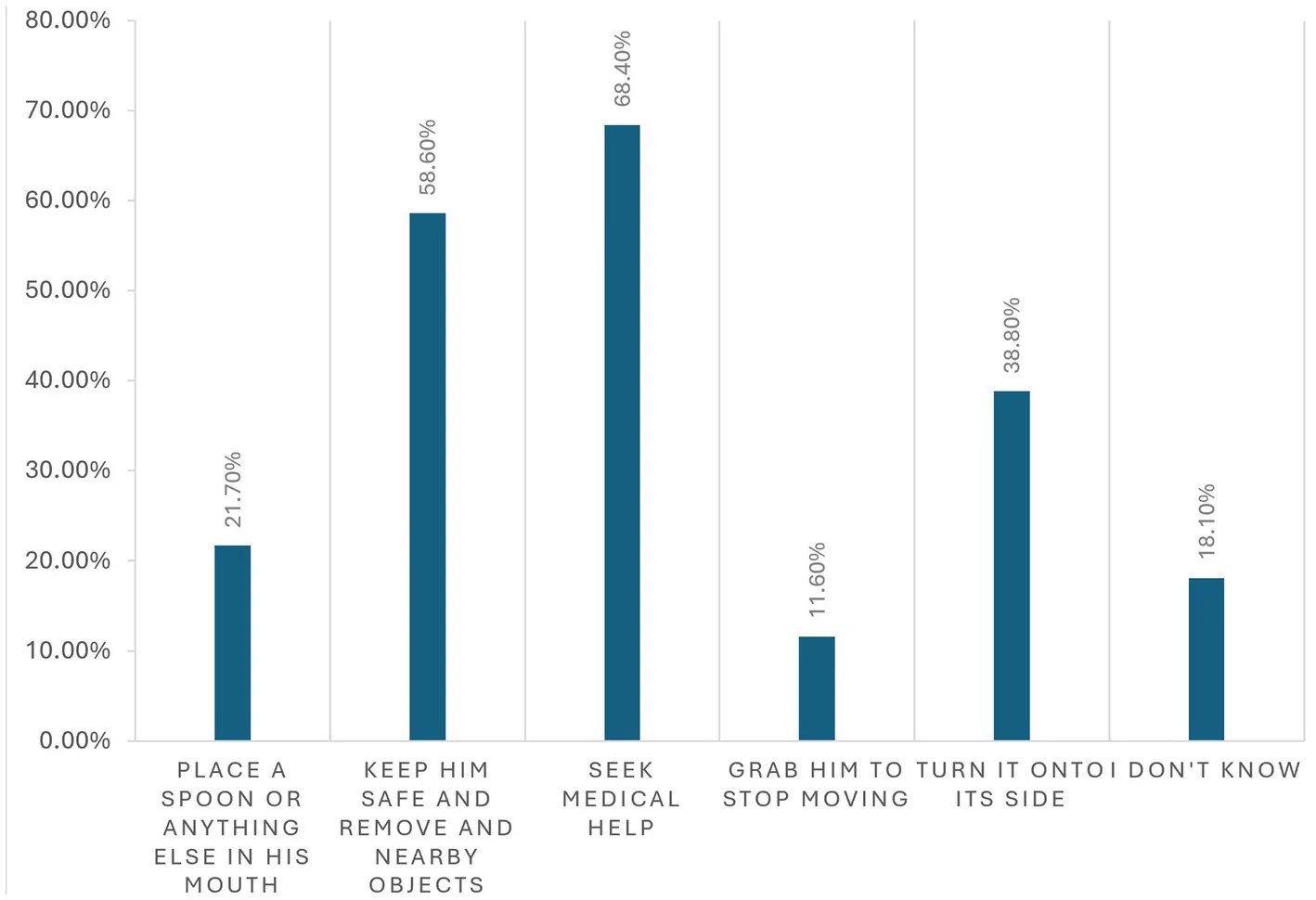
Seizure first-aid responses.

Attitudes toward childhood epilepsy were generally favorable. Most participants supported greater public education about epilepsy, interaction between their children and children with epilepsy, and the inclusion of children with epilepsy in regular schools. Most also believed that children with epilepsy could live normal lives with appropriate treatment. Support for participation in sports was lower than for the other attitude items, while few participants considered epilepsy a social stigma (Table 4).

**Table 4:** Attitudes toward epilepsy (*N* = 580).

Item and response	n (%)
Is there a need for more public education about epilepsy?	
No	71 (12.2%)
Yes	509 (87.8%)
Would you allow your child to play with a child who has epilepsy?	
No	72 (12.6%)
Yes	498 (87.4%)
Do you think children with epilepsy can succeed in a normal school environment?	
No	57 (9.9%)
Yes	518 (90.1%)
Do you think children with epilepsy can live normal lives if they are treated properly?	
No	16 (2.8%)
Yes	559 (97.2%)
Should children with epilepsy be allowed to participate in sports?	
No	159 (27.7%)
Yes	416 (72.3%)
Do you believe that epilepsy is a social stigma?	
No	544 (94.8%)
Yes	30 (5.2%)

Statistical analyses showed several significant demographic differences in knowledge, awareness, and attitudes (Table 5). Females scored significantly higher on knowledge than males. Knowledge dropped significantly as age increased. Those aged 18 to 30 scored the highest, while participants over 60 scored the lowest. Educational level was significantly associated with all three domains, with participants holding a master’s degree or higher having the highest mean ranks for knowledge and awareness. Previous exposure to epilepsy was significantly associated with higher knowledge, awareness, and attitude scores. Nationality was significantly associated with attitudes with Saudi participants having higher attitude scores than non-Saudi participants. Monthly income and marital status showed some links to knowledge, though the marital status result requires caution due to the small divorced or widowed sample size (*n* = 14).

**Table 5:** Mean ranks for knowledge, awareness, and attitude by demographic variables.

Group	Knowledge MR	Awareness MR	Attitude MR
Nationality			
Saudi	292.95	291.08	296.88
Non-Saudi	269.99	285.63	237.21
*p*-value	0.304	0.802	0.004
Gender			
Female	305.83	290.74	295.64
Male	267.45	286.77	280.50
*p*-value	0.006	0.769	0.233
Exposure			
No	160.23	44.18	232.38
Yes	299.38	307.28	294.46
*p*-value	<0.001	<0.001	0.016
Age			
18–30	311.99	275.75	281.44
31–45	290.79	295.51	307.56
46–60	263.22	313.39	303.20
>60	222.88	290.45	231.77
*p*-value	0.004	0.149	0.050
Marital status			
Single	310.13	277.74	276.69
Married	269.39	298.25	301.17
Divorced/Widowed	347.07	284.82	238.50
*p*-value	0.006	0.322	0.073
Education level			
Elementary or below	147.55	220.77	199.36
High school	284.66	287.10	277.53
Diploma	235.94	282.91	257.17
Bachelors	308.68	287.37	310.30
Master’s or above	335.50	357.18	305.15
*p*-value	<0.001	0.049	0.008
Monthly income (SAR)			
<3,000	324.05	265.64	299.53
3,000–7,999	291.99	293.01	284.25
8,000–14,999	256.04	290.40	271.61
>15,000	291.25	307.88	308.32
Prefer not to say	271.65	287.34	275.46
*p*-value	0.018	0.400	0.297

Mann–Whitney U tests were used for Nationality, Gender, and Exposure comparisons. Kruskal-Wallis H tests were used for Age, Marital Status, Education Level, and Monthly Income comparisons.

Correlation analyses showed significant positive, although weak, associations between all three variables (Table 6). Knowledge had a significant positive relationship with awareness (*r* = 0.31, *p* < 0.001) and attitudes (*r* = 0.24, *p* < 0.001). Awareness also had a significant positive relationship with attitudes (*r* = 0.23, *p* < 0.001).

**Table 6:** Spearman correlations between knowledge, awareness, and attitude.

Variable	1	2	3
Knowledge	—	0.31** (<0.001)	0.24** (<0.001)
Awareness	0.31** (<0.001)	—	0.23** (<0.001)
Attitude	0.24** (<0.001)	0.23** (<0.001)	—

**Values are statistically significant at *p* < 0.001.

The multivariable logistic regression analyses identified several factors associated with knowledge awareness and attitudes after adjustment for sociodemographic characteristics. For knowledge, higher educational attainment was significantly associated with greater odds of good knowledge. Participants holding a bachelor’s degree had about 13 times the odds of good knowledge compared with those with elementary education or below, while those with a master’s degree or higher had about 16 times the odds. Gender was also independently associated, with knowledge with males having roughly half the odds of females. Previous exposure to epilepsy was associated with nearly fourfold higher odds of good knowledge after adjustment for sociodemographic characteristics. The knowledge model explained approximately 12% of the variance (Table 7).

**Table 7:** Binary logistic regression predicting knowledge about epilepsy (*N* = 580).

Predictor	B	*p*	aOR [95% CI]
Nationality (Non-Saudi)	−0.08	0.791	0.92 [0.50, 1.69]
Age group			
18–30 years (ref.)	—	—	—
31–45 years	−0.02	0.970	0.99 [0.45, 2.17]
46–60 years	−0.27	0.513	0.76 [0.34, 1.72]
> 60 years	−0.19	0.737	0.83 [0.27, 2.50]
Gender (Male)	**−0.65**	0.**002**	**0.52 [0.35, 0.78]**
Marital status			
Single (ref.)	—	—	—
Married	−0.37	0.333	0.69 [0.32, 1.47]
Divorced/Widowed	−0.12	0.870	0.89 [0.22, 3.56]
Educational level			
Elementary or below (ref.)	—	—	—
High school	**1.87**	0.**020**	**6.46 [1.34, 31.22]**
Diploma	**2.15**	0.**011**	**8.62 [1.64, 45.40]**
Bachelor’s	**2.57**	0.**002**	**13.06 [2.63, 64.83]**
Master’s or above	**2.78**	0.**002**	**16.18 [2.87, 91.22]**
Monthly income (SAR)			
< 3,000 (ref.)	—	—	—
3,000–7,999	0.01	0.973	1.01 [0.48, 2.15]
8,000–14,999	−0.56	0.106	0.57 [0.29, 1.13]
> 15,000	0.03	0.935	1.03 [0.50, 2.12]
Prefer not to say	−0.41	0.108	0.66 [0.40, 1.09]
Exposure to epilepsy (Yes)	**1.30**	**<0.001**	**3.68 [1.73, 7.83]**
Constant	−2.35	0.008	0.10

Model Statistics −2 Log Likelihood = 720.22, Cox & Snell R^2^ = 0.092, Nagelkerke R^2^ = 0.123, Hosmer–Lemeshow χ^2^(8) = 13.164, *p* = 0.106. Bold values indicate statistically significant associations (*p* < 0.05).

For awareness, having a master’s degree or above was associated with higher odds of good awareness compared with elementary education or below (aOR 5.53, 95% CI 1.19–25.65). No other demographic variables were significantly associated with awareness. The estimate for previous exposure to epilepsy was unstable, with an extremely large non-significant coefficient. The model overall explanatory power was similar to the knowledge model at around 14% of variance explained (Table 8).

**Table 8:** Binary logistic regression predicting awareness about epilepsy.

Predictor	B	*p*	aOR [95% CI]
Nationality (Non-Saudi)	0.04	0.896	1.04 [0.58, 1.88]
Age group			
18–30 years (ref.)	—	—	—
31–45 years	0.47	0.245	1.60 [0.72, 3.54]
46–60 years	0.64	0.127	1.89 [0.83, 4.28]
> 60 years	0.86	0.132	2.36 [0.77, 7.24]
Gender (Male)	−0.11	0.581	0.89 [0.60, 1.33]
Marital status			
Single (ref.)	—	—	—
Married	−0.25	0.515	0.78 [0.37, 1.66]
Divorced/Widowed	0.01	0.985	1.01 [0.27, 3.80]
Educational level			
Elementary or below (ref.)	—	—	—
High school	0.98	0.163	2.65 [0.67, 10.43]
Diploma	0.99	0.187	2.68 [0.62, 11.54]
Bachelor’s	0.87	0.222	2.38 [0.59, 9.55]
Master’s or above	**1.71**	0.**029**	**5.53 [1.19, 25.65]**
Monthly income (SAR)			
< 3,000 (ref.)	—	—	—
3,000–7,999	0.04	0.917	1.04 [0.50, 2.17]
8,000–14,999	−0.01	0.969	0.99 [0.51, 1.92]
> 15,000	0.19	0.594	1.21 [0.60, 2.46]
Prefer not to say	0.09	0.704	1.10 [0.68, 1.79]
Exposure to epilepsy (Yes)	21.24	0.997	1.67 × 10^9^ [0.00, —]
Constant	−22.36	0.997	—

Model Statistics −2 Log Likelihood = 720.77, Cox & Snell R^2^ = 0.108, Nagelkerke R^2^ = 0.144, Hosmer–Lemeshow χ^2^(8) = 2.63, *p* = 0.956. Bold values indicate statistically significant associations (*p* < 0.05).

For attitudes, fewer variables reached statistical significance. Being male was associated with less favorable attitudes, reducing the odds by about one-third compared to females. Interestingly, those in the middle-income bracket of 8,000–14,999 SAR showed significantly less favorable attitudes compared to the lowest income group. Exposure to epilepsy again emerged as significant, doubling the odds of positive attitudes. However, the attitude model explained less variance overall, with a Nagelkerke R^2^ of only 8%, suggesting that attitudes may be influenced by factors not captured in this analysis. All three models showed no evidence of poor fit based on the Hosmer-Lemeshow tests (Table 9).

**Table 9:** Binary logistic regression predicting attitude.

Predictor	B	*p*	aOR [95% CI]
Nationality (Non-Saudi)	−0.54	0.074	0.58 [0.32, 1.05]
Age group			
18–30 years (ref.)	—	—	—
31–45 years	0.15	0.702	1.16 [0.54, 2.50]
46–60 years	0.11	0.778	1.12 [0.51, 2.48]
> 60 years	−0.55	0.321	0.58 [0.19, 1.72]
Gender (Male)	−0.41	0.038	0.66 [0.45, 0.98]
Marital status			
Single (ref.)	—	—	—
Married	0.27	0.462	1.32 [0.64, 2.72]
Divorced/Widowed	−0.38	0.563	0.68 [0.19, 2.48]
Educational level			
Elementary or below (ref.)	—	—	—
High school	0.86	0.227	2.35 [0.59, 9.42]
Diploma	0.86	0.251	2.37 [0.54, 10.32]
Bachelor’s	1.26	0.079	3.53 [0.87, 14.42]
Master’s or above	0.87	0.265	2.39 [0.52, 11.01]
Monthly income (SAR)			
< 3,000 (ref.)	—	—	—
3,000–7,999	−0.19	0.600	0.83 [0.40, 1.69]
8,000–14,999	−0.74	0.028	0.48 [0.25, 0.92]
> 15,000	−0.10	0.772	0.90 [0.45, 1.81]
Prefer not to say	−0.45	0.066	0.64 [0.40, 1.03]
Exposure to epilepsy (Yes)	0.72	0.046	2.06 [1.01, 4.19]
Constant	−1.22	0.124	—

Model Statistics: −2 Log Likelihood = 751.88, Cox & Snell R^2^ = 0.058, Nagelkerke R^2^ = 0.077, Hosmer–Lemeshow χ^2^(8) = 11.06, *p* = 0.198.

## Discussion

This cross-sectional study assessed knowledge, awareness, and attitudes toward childhood epilepsy among residents of Al Madinah Al Munawwara and examined the sociodemographic factors associated with these domains. The findings showed high general awareness of epilepsy and favorable attitudes toward children with the condition. However, important gaps remained in knowledge, especially in relation to seizure management and first aid. Higher educational attainment and previous exposure to epilepsy were associated with better knowledge, while previous exposure was also associated with more favorable attitudes.

Most participants had previously heard or read about epilepsy, which is consistent with earlier studies from Saudi Arabia. A survey conducted in five regions of Saudi Arabia found high awareness of epilepsy but reported misconceptions about its causes (8). Likewise, a study of 2,626 residents of the Al Madinah region reported high familiarity with epilepsy despite moderate levels of knowledge (13). The present study showed a similar pattern, with over 30% of participants considering epilepsy a mental illness and a lifelong disease. These findings show that familiarity with epilepsy does not necessarily reflect an accurate understanding of the condition.

The main knowledge gap concerned seizure management and first aid. Around two-thirds of participants reported that they did not know what to do during a seizure or how to provide first aid to a child experiencing one. Similar findings have been reported in Al Madinah City where only 32% of participants demonstrated good overall knowledge of epilepsy and many lacked knowledge of appropriate actions during a seizure (20). Poor seizure first aid knowledge has also been reported among undergraduate health students in Riyadh (21) and teachers in Makkah (22). This suggests that limited first aid knowledge is not restricted to people without previous health education and supports greater emphasis on practical seizure management in epilepsy education.

Some participants also selected inappropriate first aid actions such as placing an object in the child’s mouth or restraining the child during a seizure. Similar misconceptions about seizure management have been reported in other settings (23, 24). The belief that an object should be placed in the mouth during a seizure remains common (25) and misinformation about seizure first aid has also been reported on social media platforms (26). Public education should therefore address specific first aid misconceptions and teach appropriate actions during a seizure, rather than focus only on general information about epilepsy. Such interventions could include brief community sessions, school-based education for teachers and parents, first aid demonstrations, and simple educational materials distributed through social media and primary healthcare settings.

Education was strongly associated with knowledge. Participants with higher levels of education had greater odds of good knowledge compared with those with elementary education or below. Higher educational level was also associated with better epilepsy knowledge in previous studies conducted in Saudi Arabia (13, 15, 20, 27). A Palestinian community study also found higher education to be associated with better awareness and more favorable attitudes toward epilepsy (28). These findings support the need for educational materials that are accessible to people with different levels of formal education, using simple language, visual demonstrations, and practical examples.

Male participants had lower odds of good knowledge and favorable attitudes compared with females. Previous studies have also reported gender differences in epilepsy knowledge and awareness with better knowledge among females in Al Madinah (20), and lower awareness among males in another community-based study (28). Differences in exposure to health information or caregiving roles might contribute to this association, although these factors were not assessed in the present study. Further research is needed to understand the reasons for these differences and whether educational approaches should address gaps among specific groups.

Previous exposure to epilepsy was associated with better knowledge and more favorable attitudes after adjustment for demographic factors. This is consistent with several studies that found social contact with people with epilepsy is associated with better knowledge and lower levels of stigma (29–31). Familiarity through personal contact may contribute to a better understanding of epilepsy and reduce misconceptions (6). This finding may inform future interventions by supporting opportunities for meaningful contact with people with epilepsy as part of educational and awareness programs.

The correlation analysis showed that knowledge, awareness, and attitudes were positively correlated, although the associations were weak. This suggests some interrelationship between these constructs, although the direction of these relationships cannot be assumed. Evidence from epilepsy studies indicates that improvements in knowledge do not always correspond to comparable changes in attitudes (32, 33). This supports assessing knowledge and attitudes separately when evaluating community education programs rather than assuming that improvement in one will result in improvement in the other.

Despite the identified gaps in knowledge most participants expressed supportive views toward children with epilepsy. Almost 95% did not perceive epilepsy as a social stigma, although social desirability may have influenced responses. A recent largescale Saudi study reported a similar pattern with most participants rejecting the view that people with epilepsy are inferior, yet almost half opposed marriage to a person with epilepsy and nearly one-quarter opposed their children associating with them (8). In the present study, only 12.6% opposed their child playing with a child with epilepsy, a lower proportion than reported in the national study (8), yet 27.7% did not support children with epilepsy participating in school activities. This reluctance may reflect limited knowledge or concerns about seizure-related safety rather than negative attitudes alone. Nevertheless, the willingness of 65% of participants to attend an epilepsy awareness program presents an opportunity for community education focused on the gaps identified in this study.

### Limitations

Several limitations should be considered when interpreting these findings. The cross-sectional design does not allow conclusions about causal or temporal relationships between sociodemographic factors, previous exposure to epilepsy, and the study outcomes. Convenience sampling and online recruitment may limit the representativeness of the sample and reduce the generalizability of the findings to the wider population of Al Madinah Al Munawwara. Self-reported responses are also subject to recall and social desirability bias, especially for questions related to attitudes and stigma. The Cronbach’s alpha values indicated modest internal consistency for the knowledge domain and low internal consistency for the awareness and attitude domains. These estimates were derived from a small pilot sample, and the domains included items addressing different aspects of knowledge, awareness, and attitudes, which may partly explain the limited internal consistency. The overall scores and associated analyses should therefore be interpreted with caution. In addition, the study assessed knowledge of seizure first aid but did not evaluate practical skills. Future studies should further assess the validity and reliability of the questionnaire in larger samples and use objective or scenario-based methods to assess seizure first-aid skills.

## Conclusion

The findings of this study indicate that residents of Al Madinah Al Munawwara have high familiarity with epilepsy and positive attitudes toward children with the condition. Despite this, substantial gaps remain in understanding seizure management and appropriate first aid responses Knowledge differed according to educational attainment and previous exposure to epilepsy, which highlights the importance of considering these factors in future educational efforts. Community programs should focus on practical seizure first aid, address common misconceptions, and provide clear information suitable for different educational levels. Schools, healthcare settings, community programs, and social media may serve as effective channels for improving public knowledge and appropriate responses to childhood seizures.

## Data Availability

The raw data supporting the conclusions of this article will be made available by the authors, without undue reservation.
