# Supplementary material for: Knowledge, attitudes, and seizure first aid for childhood epilepsy among the Madinah community, Saudi Arabia
Source: Front Public Health. 2026 Sep 21;14:1858918. doi: 10.3389/fpubh.2026.1858918 (PMC13635122; doi:10.3389/fpubh.2026.1858918)
Supplement: Supplementary file 1 [file Data_Sheet_1.PDF]

Supplementary Material

Supplementary Table S1: Cronbach's Alpha for Knowledge, Awareness, and Attitude Scales

Scale	Cronbach's α	Number of Items
Knowledge	.60	13
Awareness	.36	5
Attitude	.38	6
